# A Report of the Curriculum Task Force of the ISCB Education Committee

**DOI:** 10.1371/journal.pcbi.1002570

**Published:** 2012-06-28

**Authors:** Lonnie R. Welch, Russell Schwartz, Fran Lewitter

**Affiliations:** 1 School of Electrical Engineering and Computer Science, Ohio University, Athens, Ohio, United States of America; 2 Department of Biological Sciences and Lane Center for Computational Biology, Carnegie Mellon University, Pittsburgh, Pennsylvania, United States of America; 3 Bioinformatics and Research Computing, Whitehead Institute, Cambridge, Massachusetts, United States of America; University of California San Diego, United States of America

## Introduction

The International Society for Computational Biology (ISCB) Education Committee (EduComm) promotes worldwide education and training in computational biology and bioinformatics and serves as a resource and advisor to organizations interested in developing educational programs.

The topic of curricula for bioinformatics programs has long been of interest to ISCB and EduComm. Dr. Russ Altman, a founding board member and past president of ISCB, has been associated with one of the first bioinformatics degree programs (at Stanford University) and wrote an article on this topic [1]. Dr. Shoba Ranganathan, as chair of EduComm a decade ago, began organizing a yearly Workshop on Education in Bioinformatics (WEB) at Intelligent Systems for Molecular Biology (ISMB) meetings that generated exchange of information and many productive discussions. Curriculum development was one aspect of bioinformatics education covered in these sessions [2].

The field of bioinformatics has grown in the past decade. There are many such degree granting programs around the world at the bachelor's, master's, and PhD levels. This article provides a status report of the EduComm's ongoing endeavor to identify a set of core curricular guidelines for bioinformatics education at all levels. As a pilot project, the Curriculum Task Force of the EduComm conducted a survey in the spring of 2011. This initial survey was sent to members of the EduComm, consisting of 50 individuals from various regions of the world, and to the EMBnet community, representing 79 people from more than 30 countries. The response rate was 33%, with 41 individuals completing the survey. Analysis of the survey produced an initial set of recommendations to be used as a discussion point from which to launch a larger effort to develop a working bioinformatics curriculum. With increased input from the larger community, the EduComm will continue to refine its results. Individuals who are interested in contributing to this initiative are encouraged to contact the Chairs of the ISCB EduComm.

The purposes of this article are to further disseminate the survey results and to solicit participation in the initiative. The initial survey results are summarized, the preliminary working curriculum is defined, and the next steps of the EduComm Curriculum Task Force are outlined.

## Survey Results

Responses were received from 41 individuals in 20 countries (covering five continents). This is a small but diverse group of respondents representing a wide array of professional positions, including scientist, professor (all ranks), director of bioinformatics, technician, engineer, postdoctoral researcher, teaching assistant, and lecturer. The levels of students taught by the respondents ranged from secondary thru PhD.

Topics suggested by survey respondents for inclusion in a bioinformatics curriculum fit into two primary areas, (1) *computation, mathematics, and statistics* and (2) *biology and chemistry*. An initial working bioinformatics curriculum was constructed by selecting topics suggested by at least ten respondents (i.e., more than 25% of the survey respondents), resulting in five topics in each of the two primary areas. This initial working curriculum is shown in Table 1.

**Table 1:** The initial working bioinformatics curriculum.

Computation, Mathematics, and Statistics	Biology and Chemistry
Programming/scripting/software engineering (36)	Cellular and molecular biology (21)
Statistics/probability (31)	Genomics (12)
Databases (24)	Basic biology (11)
Algorithm design/data structures/computation theory (20)	Evolutionary biology (10)
Machine learning (13)	Genetics (10)

Each number in parentheses indicates the total number of survey respondents who recommend the corresponding topic.

## Analysis and Next Steps

The results of our survey were presented at the Third RECOMB Satellite Conference on Bioinformatics Education (RECOMB-BE) [3]. Several observations and suggestions were offered during the discussion that followed the presentation. It was noted that the survey did not provide a list of possible topics from which to choose. This was intentional, to avoid introducing biases that would affect the answers. The unrestricted nature of the questions resulted in a wide array of topics being suggested. Responses that addressed similar concepts were grouped together to identify general topics.

The initial working curriculum does not completely represent the breadth of suggested topics. For example, it does not contain anything explicitly related to medicine, structural biology, or biochemistry. A small minority of the respondents suggested these topics; thus, they are not reflected in the consensus. (Additional grouping could be performed in order to increase the coverage of the responses.) In addition, due to the diversity of suggestions, the topic categories above tend to be general areas rather than contents of specific courses. Furthermore, due to the use of the word *topic* in the survey, respondents suggested only content areas, not issues of process, which appear frequently in descriptions of desired program outcomes. Specific examples of missing items include (1) scientific communication, (2) lifelong learning, and (3) professional behavior (including ethics).

It was also suggested that ISMB's topic areas be considered as a general framework for a bioinformatics curriculum. The most recent ISMB topic areas are as follows:

- Applied Bioinformatics
- Bioimaging & Data Visualization
- Databases & Ontologies
- Disease Models & Epidemiology
- Evolution & Comparative Genomics
- Gene Regulation & Transcriptomics
- Mass Spectrometry & Proteomics
- Population Genomics
- Protein Interactions & Molecular Networks
- Protein Structure & Function
- Sequence Analysis
- Text Mining

This initial working curriculum is only intended to prompt discussion and to inspire the generation of more specific recommendations for refined curricula for bioinformatics. It provides useful guidelines for those seeking to determine core topics for a bioinformatics program (it is not intended to be used as a standard for accreditation purposes). The EduComm is currently (a) summarizing curricula from existing bioinformatics programs, (b) surveying directors of bioinformatics core facilities and biological researchers to identify the skills needed for people they hire, and (c) reviewing bioinformatics career opportunities to determine skill sets required by current employers of bioinformaticians. The new survey results may be used to propose curricular guidelines.

The ISCB EduComm (http://www.iscb.org/iscb-leadership-a-staff-/1172) invites participation from the worldwide community of computational biologists and bioinformaticians. Please join us for the Birds of a Feather (BoF) session, entitled Curriculum Guidelines for Bioinformatics and Computational Biology (An Open Forum of the Curriculum Task Force of the ISCB Education Committee), which will be held on July 16, 2012 at the ISMB meeting (http://www.iscb.org/ismb2012-program/birds-of-a-feather). At the BoF session, the Curriculum Task Force of the ISCB Education Committee will hold an open forum to discuss bioinformatics curriculum guidelines. Participants will consider curricular implications of the task force's surveys of career opportunities, hiring practices of bioinformatics core facility directors, and existing curricula. The forum seeks input from all interested individuals.

Additionally, we are seeking input via a blog. To read a more detailed report of the survey and to post your comments, please visit the blog site at http://bioinfocurriculum.blogspot.com/.
